# Small predators dominate fish predation in coral reef communities

**DOI:** 10.1371/journal.pbio.3001898

**Published:** 2022-11-29

**Authors:** Michalis Mihalitsis, Renato A. Morais, David R. Bellwood

**Affiliations:** 1 Research Hub for Coral Reef Ecosystem Functions, James Cook University, Townsville, Queensland, Australia; 2 College of Science and Engineering, James Cook University, Townsville, Queensland, Australia; 3 Australian Research Council Centre of Excellence for Coral Reef Studies, James Cook University, Townsville, Queensland, Australia; 4 Department of Evolution and Ecology, University of California, Davis, California, United States of America; Estacion Biologica de Doñana CSIC, SPAIN

## Abstract

Ecosystem processes are challenging to quantify at a community level, particularly within complex ecosystems (e.g., rainforests, coral reefs). Predation is one of the most important types of species interactions, determining several ecosystem processes. However, while it is widely recognised, it is rarely quantified, especially in aquatic systems. To address these issues, we model predation on fish by fish, in a hyperdiverse coral reef community. We show that body sizes previously examined in fish–fish predation studies (based on a metanalysis), only represent about 5% of likely predation events. The average fish predator on coral reefs is just 3.65 cm; the average fish prey just 1.5 cm. These results call for a shift in the way we view fish predation and its ability to shape the species or functional composition of coral reef fish communities. Considered from a functional group approach, we found general agreement in the distribution of simulated and observed predation events, among both predator and prey functional groups. Predation on coral reefs is a process driven by small fish, most of which are neither seen nor quantified.

## Abstract 2

When we think of fish predators on coral reefs we immediately think of sharks, groupers and barracudas. By modelling entire fish communities and comparing the results with published gut content data, this study reveals that the average fish-eating-fish predator on coral reefs is under 5 cm long; the vast majority of life and death events involve small fish being eaten by small predators.

## Introduction

For many animals and plants in high diversity systems, such as coral reefs, population dynamics are driven by early life stage mortality, with recruitment functioning as a population bottleneck [1–3]. Indeed, predation-based mortality is widely regarded as one of the most important species interactions determining fish population structures on coral reefs [4–6]. On reefs, many, if not most fishes, are eaten by other fishes (7). While the consequences of high mortality in these ecosystems is well documented [8,9], the predators that drive this process are largely unknown.

In the last few years, the main focus of fish predation studies on coral reefs has been on trophic cascades [10–13], its effects on prey abundance [14,15], or indirect “fear-effects” on prey behaviour [16–19]. The fish predators investigated in these fields were primarily sharks or other large mesopredators. However, recent work has highlighted the potential trophic importance of small-bodied coral reef fishes [20,21]. This raises questions over the identity and size of fish predators and their relative importance from an ecosystem function perspective. Basically, big questions still remain: Who are the main predators of fishes on coral reefs? At which size does most predation happen? And, while all individuals in a community go through the predation gauntlet, how do these interactions scale at a community level?

Indeed, quantifying fish predation at a community level poses some challenges. Generally, fish–fish predation has been quantified through gut content analyses [7,22,23]. However, this analysis requires a high number of specimens for a small yield of data, as most often, piscivore guts are empty [24,25]. While fish–fish predation is widely acknowledged, it is logistically challenging to quantify in situ, given the duration of these events only lasting few milliseconds [26,27]. Furthermore, many predator–prey estimates are based on length–length relationships. However, especially within coral reef fishes, body depth (and therefore a determinant of gape limitation [28]) is a primary axis of variation [29,30]; assuming predator–prey relationships based on length, risks the loss of significant variation in the data. Lastly, approaches incorporating species abundance alone, may lead to the loss of information on the ontogenetic history of fishes (for example, a juvenile predator is also prey during its early life stages), or, to incorrect estimates of interaction rates (i.e., presence does not always equate to function) [31]. Therefore, for the reasons above, there is a need to quantify predation events at a community level, using a different approach.

Given the recent advances in, and promising results from, a functional group approach [32] to the investigation of ecosystem processes [33–35], we apply this approach to coral reef fishes, with a particular focus on predation on fishes, by fishes. We first surveyed a coral reef fish community and constructed an algorithm to model predator–prey interactions based on the functional constraints imposed by both predators and prey (following [36,37]). Predators were classified by their functional group (grabbers versus engulfers) and size, while prey were classified by body depth (which determines the size of predators able to feed on them) and prey functional group (cryptobenthic, epibenthic, social) [36]. This produced a standardised surveyed community of 32,218 fish that were simulated 1 million times to produce 349,000 potential predation events (i.e., functionally viable events, by removing forbidden interactions [38,39]). These results were then compared to the documented consumption of reef fish prey by fish predators, based on a metanalysis of gut content data (*n* = 1,677 predation events) across Indo-Pacific coral reef ecosystems.

## Results

**Fig 1:**
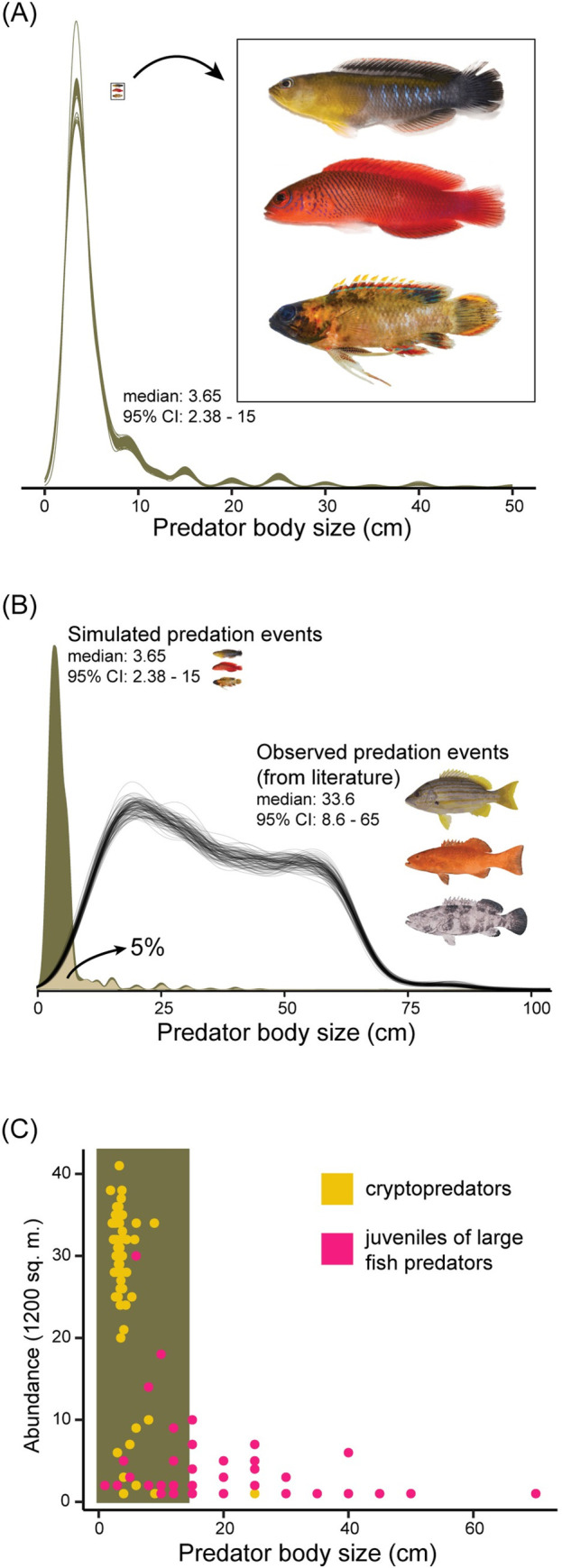
Cryptopredators, not “large” reef fishes, dominate predation events on coral reefs. (A) Community-level predation of coral reef fishes, along a predator size gradient, based on simulations from reef surveys. Examples of cryptopredators that likely shape community composition in coral reef ecosystems, top to bottom: *Pseudochromis cyanotaenia*, *Cypho purpurascens*, *Plesiops* sp. (B) Predation events simulated from our surveyed community (same as (A)) vs. observed predation events (metanalysis of literature). Multiple lines in observed predation events reflect draws from a distribution (see Materials and methods). (C) Abundance estimates of cryptopredator species, relative to juveniles of “large” reef fish predator species. The coloured box represents the size range within which most predation events are estimated. Original photographs for clipart in (A) were originally sourced from C.R. Hemingson, with permission. Original photos for clipart in (B) were taken by J.E. Randall and sourced from [40]. The data underlying this figure can be found in DOI: 10.5281/zenodo.6772154.

We found that fish predation on coral reefs is overwhelmingly dominated by small, diminutive predators. The average fish predator that feeds on other fish on reefs is just 3.6 cm, and the average prey just 1.5 cm. By combining surveys at different spatial scales, to generate as complete a census as possible, our standardised surveyed reef fish community contained 32,218 fishes from 266 species. Simulating 1 million potential predator–prey interactions (i.e., predation events) within this community, by applying size-based functional constraints (prey body depth/predator gape size), we obtained 349,081 potential (i.e., functionally feasible) predation events. In this extensive pool of potential events, the median size of a predator fish was just 3.65 cm (95% CI: 2.38 to 15) total length (TL) (mean: 5.6 cm) (Fig 1A). Essentially, 95% of potential predation events involve predators less than 15 cm. When simulated predation events from our surveyed community were compared to a literature-based dataset of 1,677 observed predation events, by size, there was only 5% overlap (Fig 1B). In essence, the vast majority of studies have exclusively quantified predation by exceptionally large predators; most predation events go unobserved and unrecorded. Our results suggest there is a need for a shift in the way we consider fish predation, and how trophic interactions shape the species, and functional, composition of coral reef fish communities.

While exponentially declining mortality rates appear to be the norm for reef fishes [8,9,41], the predators that drive these curves have remained largely unknown. Our results emphasize the small size of these predators. Furthermore, based on abundance-based encounter likelihoods, these predators are unlikely to be juveniles of “large” reef fish predators; the predators driving the process are predominantly cryptopredators (Fig 1C), defined herein as carnivorous fishes below 10 cm.

**Fig 2:**
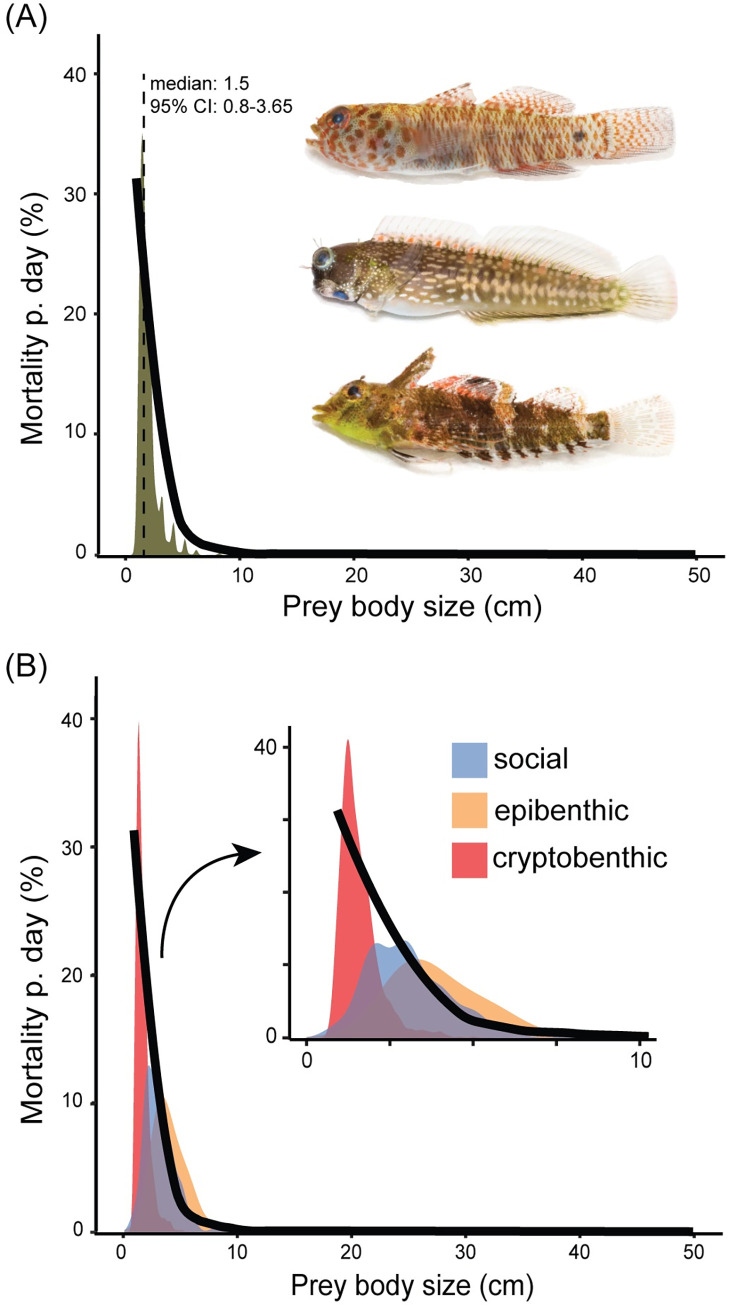
Cryptobenthic fishes are the main prey fishes on coral reefs. (A) Community level predation simulated in our study along a prey size gradient (brown), mirrors the exponentially declining line that represents observed reef fish mortality rates from an independent metanalysis on reef fish mortality [8]. Examples of primary contributors to this density distribution, from top to bottom: *Eviota queenslandica*, *Salarias alboguttatus*, *Enneapterygius tutuilae*. (B) The same density curve, in 2A, split according to prey functional groups, namely: cryptobenthic = red, epibenthic = yellow, social = blue. Original photographs for clipart in (A) were originally sourced from C.R. Hemingson, with permission. The data underlying this figure can be found in DOI: 10.5281/zenodo.6772154.

The same patterns apply to prey fish. The estimated median size of prey fish was just 1.5 cm TL (95% CI: 0.8 to 3.65) (mean: 1.75 cm) (Fig 2A); 95% of potential predation events involve prey sizes less than 3.65 cm. Functionally feasible predation events were simulated based on prey body depth versus gape size relationships. These simulations resulted in 349,081 functionally feasible predation events. Of these events, the prey involved were: 90.4% cryptobenthic substratum dwellers (referred to hereafter as cryptobenthic), 8.4% social prey, and 1.2% solitary epibenthic (referred to as epibenthic) (Fig 2B) (for details on functional groups, see S1 Table). When these predation events are compared to values of published reef fish mortality rates, their distribution matches closely (Fig 2).

### Functional group contributions

When the 32,218 fishes in our community were classified into prey functional groups, 59% were cryptobenthic, 7% were epibenthic, and 34% were social. Furthermore, of the 32,218 fishes in our community, 1,726 (5.4%) were considered potential fish predators (based on their trophic status from the literature, see Materials and methods). The functional groups of these predators were 85% grabbers and 15% engulfers.

### Comparing size-specific simulated predation to observed predation events at a community level

**Fig 3:**
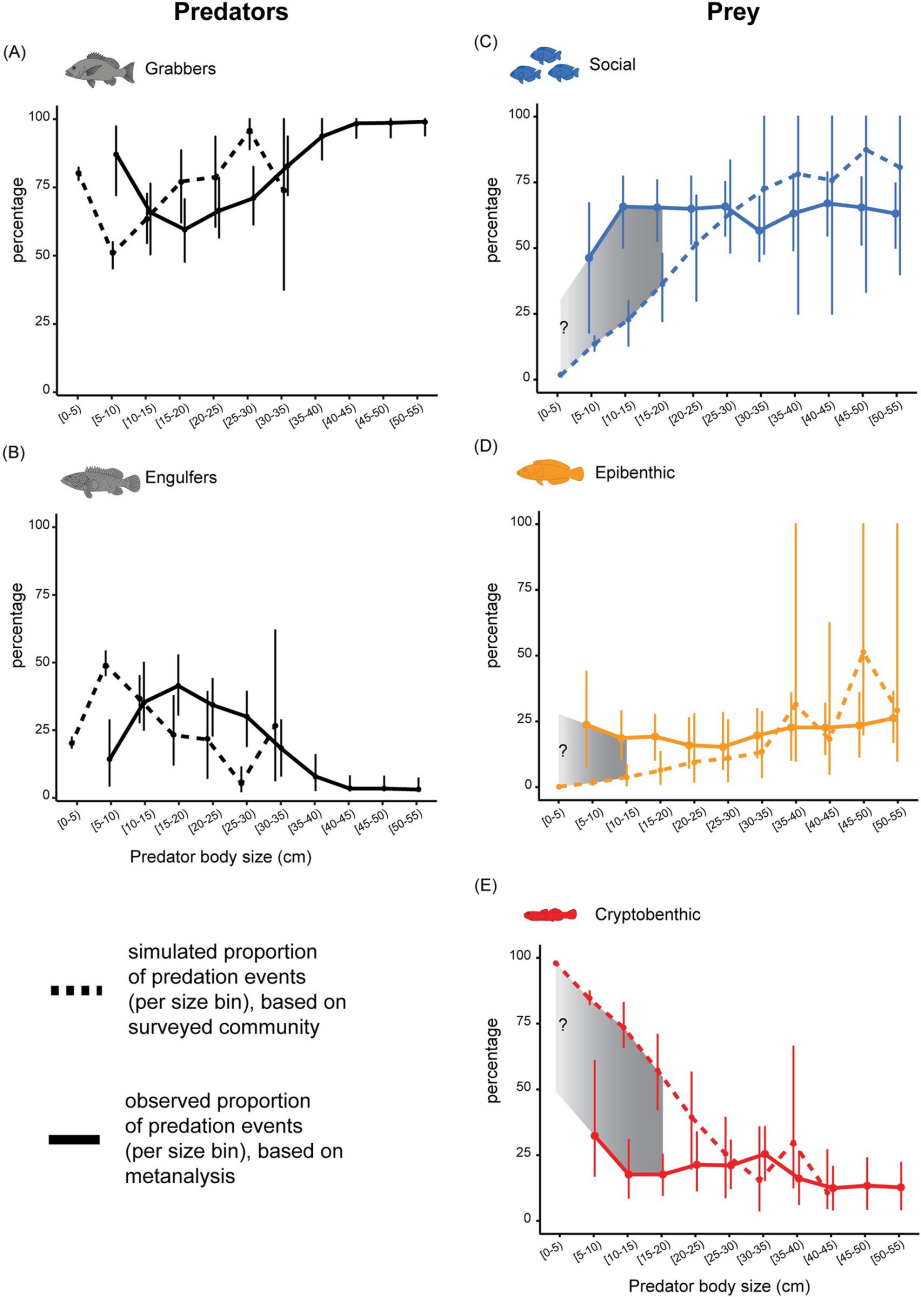
Functional groups of predator–prey fishes reveal patterns of community-level predation. Simulated vs. observed relative contributions to the process of piscivory on reefs, based on predator functional groups (A, B) and prey functional groups (C–E). Overall, the trajectories of simulated contributions (dashed lines) and observed contributions (solid lines) were in agreement for predator and prey functional groups. Disparity was only found between the 2 estimates for small predator sizes, when results are based on prey functional groups: Social and epibenthic prey were overrepresented in the diet of small predators, whereas cryptobenthic prey were underrepresented. Filled circles indicate means, whereas vertical bars indicate the range of values (minimum, maximum) for a specific size bin. Grey zones indicate size bins where a difference between predicted and observed relative contribution was found to be significant. Fish silhouettes redrawn from [36] and [37]. The data underlying this figure can be found in DOI: 10.5281/zenodo.6772154.

The distribution of potential predation events among different size classes of predators in our simulated community, was found to closely reflect the distributions in our metanalysis. This applied to both predator functional groups (grabbers and engulfers) (Fig 3A and 3B) and all 3 prey functional groups (Fig 3C–3E), except for small predator body sizes. In essence, for small predators (i.e., ≤20 to 25 cm), cryptobenthic prey are underrepresented (Fig 3E), while epibenthic and social prey are overrepresented (Fig 3C–3E). This may be linked to the functional traits of these prey groups and the predators involved in this predation.

## Discussion

Cryptopredators have only recently been identified as significant fish consumers [21]. Their overwhelming abundance (relative to juveniles of large reef fish predators), along with our results of simulated community-level predation, highlight the potential of these previously overlooked cryptopredators to be the primary contributors to the process of fish predation in coral reef ecosystems. Common examples of cryptopredators (cryptobenthic reef fishes sensu Brandl, Goatley [42] which are carnivorous) include the Pseudochromidae, Plesiopidae, Gobiidae, and Apogonidae. Most of the species within these families remain under 15 cm throughout their lives [42].

The fishes consumed by cryptopredators, based on our simulations, are overwhelmingly cryptobenthic (approx. 90% of predation events) (Fig 2). Our results mirror previous empirical studies showing a high consumption of, and high turnover in, cryptobenthic fishes; a “crypto-pump,” fuelling coral reef ecosystems [20]. In essence, our results illuminate the “dark-productivity” (sensu 20) that fuels coral reefs, by identifying their most likely predators. These fishes sustain some of the most important trophic pathways on coral reefs (e.g., the detrital and piscivory-cryptobenthic pump). This may add to the variety of mechanisms of energy recycling, which appear to be an essential attribute of oligotrophic ecosystems with high species diversity and biomass [43,44]. We show that one of the key pathways that links these fishes to the rest of the food-chain is through cryptopredators.

Although the underestimation of cryptobenthic prey in the diet of predators may be associated with methodological challenges such as high digestion rates and visual identification of prey in guts [20,45], we suggest that there may also be underlying reasons associated with the specific features of this prey functional group (cryptobenthic prey). There is overwhelming evidence, that most mortality in fishes occurs during the early life stages at small body sizes, and that this is due to predation [6,8,46,47]. This is at odds with the life history of cryptobenthic fishes. How can cryptobenthic fishes, the shortest living vertebrates [48,49], with extremely small body sizes and extremely high mortality rates, maintain viable populations? Sustained temporal reproduction [50], fast growth [51], and abundant larvae [20] certainly all help to facilitate the extreme cryptobenthic lifestyle. However, our data strongly suggest that their success may also be dependent on their ability to reduce relative predation risk below what would be expected based on their size alone. By reducing predation, they would be better able to spread predation-based mortality throughout their life on the reef, sustaining a higher number of reproducing individuals during this vulnerable period (S2 Fig). These benefits may be directly related to the characteristics of the cryptobenthic functional prey group such as the behaviour of “sitting” on the benthos [36] and cryptic colouration [52]. The drab colouration and “sitting still” may indeed be a highly successful antipredatory strategy.

The results discussed above, only became evident when investigating the community from a functional group perspective. Our simulation approach allows us to make estimates on the sizes of predators and prey involved, as well as the potential relationships between the predator and prey groups. Such results have key implications for our understanding and role of functional groups within their habitat, something which has been shown for other ecosystems as well [53–55]. Indeed, we show that the functional group approach is a powerful tool in elucidating the complexities of hyperdiverse systems such as coral reefs [56–58]. This functional approach may indeed, explain how cryptobenthic prey are able to exist after all.

It is important to note that other aspects of coral reef environments (e.g., habitat complexity, exposure) [59–61] may shape predator–prey interactions for cryptopredators. Specifically, the fractal dimension/scale [62] at which these predation events occur are likely not the same as for large predatory fishes. For large predatory fishes (e.g., Lutjanidae or Serranidae), the presence of large coral structures are likely to determine the balance between escape and susceptibility to predation [63]. However, for predation by small-bodied and inconspicuous predators (i.e., cryptopredation), it is likely that coral rubble (or small corals), instead, may be the structures creating the same advantages or disadvantages, as large coral structures for larger predators. Previous studies have found a high abundance of cryptobenthic fishes in rubble areas (as opposed to coral-covered areas) [60,61,64], suggesting an intricate matrix taking place at a smaller scale, where habitat may influence predation between cryptopredators and their prey [65]. Our results may help to put rubble habitats in the context of habitat complexity and the fractal dimension [62] for the fishes living within these areas. In essence, there is a need to further investigate the influence of abiotic variables on functional traits relating to cryptopredation.

Overall, we show that the vast majority of fish predation events on coral reefs is likely to involve predators below 15 cm. The vast majority of prey in these predation events is below 5 cm. “Typical” predators on reefs, such as jacks, barracudas, and groupers, are not the ones carrying out most predation on reefs. Most fish are eaten by cryptopredators on the reef. We highlight the overwhelming importance of cryptopredators as drivers of predation at a community level. Furthermore, our data suggests that, contrary to expectations, a small body size may indeed function as an antipredatory mechanism if it is associated with “sitting” on the benthos. Our functional groups approach revealed that predation events are also governed by prey functional traits. Overall, predation on coral reefs is a game of small fishes, and cryptobenthic prey fishes appear to be winning the game.

## Materials and methods

Quantifying ecosystem processes at a community level is a logistically difficult and time-consuming process. Usually, species interactions are inferred based on collected empirical data, most often recorded as presence/absence or abundance data, or through simulation-based approaches. Here, we compare and contrast these 2.

### Quantifying predator abundance and prey availability at a community level

We first surveyed a coral reef fish community at Lizard Island, a marine reserve with no fishing, located on the Great Barrier Reef, Australia, following [66]. Fish surveys were conducted in all traditionally recognised reef zones (back, flat, crest, slope). Both underwater visual surveys and enclosed clove oil stations were used to maximise the proportion of the fish community surveyed. The 2 approaches were chosen for different groups of fishes, based on their body sizes and behaviours [67]. Body sizes were estimated by a single diver during visual surveys for larger-bodied fishes and were measured in the laboratory under a stereomicroscope for smaller-bodied fishes (see below). Visual surveys were done with a diver initially conducting a 50 × 5 m transect tape survey to count large (>25 cm TL), water column-positioned or fast swimming fishes likely to be scared away by the diver. Upon return along the tape, the same diver conducted a 30 × 5 m survey targeting smaller-bodied fishes that are less mobile. The diver then conducted another 30 × 5 m survey over the same area to count small-bodied, non-cryptic fishes usually found just above the reef benthos. Finally, the diver conducted a last 30 × 1 m survey to count cryptic individuals (e.g., within or under crevices) that would not have been surveyed using traditional visual surveying techniques [66]. In addition, to provide more accurate abundance estimates of cryptobenthic reef fishes, a set of 8 enclosed clove oil stations (following [67]) were deployed in each habitat. A total of 3 sets of visual surveys and 8 clove oil stations were conducted in each reef zone at each of the sites (*n* = 3 sites). For more detailed information on sampling methods, see [66]. Fish surveys were conducted in accordance with the James Cook University Ethics Committee (A2375) and the Great Barrier Reef Marine Park Authority (G17/38142.1).

To account for the different spatial extents of the different surveying methods, a resampling algorithm was constructed. This allowed scaling the observed fish abundance to a standardised common area among surveys. This procedure generated 1 standard, 1,200 m^2^ community, which can be interpreted as a reef section spanning the different reef zones, and with equal area in each of these zones. This “multihabitat” coral reef fish community had 32,235 fish individuals from 266 species. We then assigned all fishes to their respective prey functional group (based on their functional traits, see S1 Table) following [36].

Our quantification of potential predation events at a community level, started with a community dataset including fish species and body size. Based on body size and previously published relationships between body size and functional traits of both predator and prey fishes [36,37], we converted body sizes to functional trait values directly related to predator–prey interactions (i.e., prey body depth, predator gape size). We then conducted repeated simulations of potential predation events by sampling individuals (1 predator and 1 prey at a time) within the community. Only realistic interactions, based on the functional trait relationships, were considered (e.g., if the prey could fit in the predators’ gape), thus taking into account the presence of forbidden links [38,39]. Following simulations, we compared our results of potential predation, to observed consumption patterns, based on a metanalysis of the gut contents of coral reef fish predators.

The implementation of a functional group approach (based on functional traits) has reduced the initial complexity of coral reef piscivores [37,68,69]. Recent studies have identified 2 functional groups of predators, grabbers and engulfers, which differ in their morphology, striking, capturing, and prey processing behaviour [37]. Reef fish prey can also be divided into cryptobenthic substratum dwellers (referred to herein as “cryptobenthic”), solitary epibenthic (“epibenthic” herein), and social fishes, which differ in antipredatory morphological and behavioural traits, as well as in habitat use (e.g., position in water column) [36]. We note here that the term “cryptobenthic” is slightly different than that of Brandl, Goatley [42]. For a detailed description of these functional groups, see S1 Table.

Each individual fish species was assigned as a predator if that species has been found to feed on elusive prey in the literature or other online sources (e.g. 40). All fishes were considered as potential prey. Prey body size was then transformed to body depth based on the functional group of the species following [36].

$$D_{f,i}=a_{f}+b_{f}*\log\left(L_{f,i}\right),$$

where *f* is 1 of the 3 functional groups, and *i* an index denoting an individual fish,

*a_f_* = (−1.58, −0.78, −0.9), *b_f_* = (1.04, 0.74, 0.95), for *f* = (*cryptobenthic, epibenthic, social*), respectively. L is the total length of an individual fish. Predator body sizes were then transformed to gape sizes following relationships obtained from coral reef fish specimens [28,70,71], based on the functional group to which the surveyed predator belonged to:

$$G_{f,i}=a_{f}+b_{f}*L_{f,i},$$

where *f* is 1 of the 2 functional groups, and *i* an index denoting an individual fish,

*a_f_* = (0.93, 0.04), *b_f_* = (0.17,0.17), for *f* = (*engulfers, grabbers*), respectively. L is the total length of an individual fish.

We then conducted a series of simulations, whereby an individual predator from the community (along with its respective functional trait values) was randomly matched against an individual prey fish (along with its respective functional trait values). Each simulation consisted of 10,000 potential piscivory events, and the simulation was conducted 100 times with repetition. We then calculated the relationship between predator gape size and prey body depth, for each potential predation event (following 28), by dividing prey body depth to predator gape size. We only kept instances in which the obtained ratios were within the range of 0.14 to 0.7, as this has been found to be the relative prey size within which 95% of predation occurs [37], and eliminated any other instances (i.e., forbidden links [38,39]). Next, we binned these events into respective size bins of predators (from 5 to 50 cm, at 5 cm intervals), and calculated the relative contribution of each prey functional group to the overall prey availability for each predator size bin:

$$C_{f,b}=\frac{A_{f,b}}{\sum_{f=1}^{n}A_{f,b}},$$

where C is the relative contribution of available prey of a specific functional group *f* at a specific size bin *b*, *A* is the abundance of individuals of the specific prey functional group, for the given size bin, and the denominator is the summation of the abundances of all *n* functional groups. These contributions were then compared to the observed consumption of each prey functional group based on the metanalysis of gut content data (see below).

### Observed diet of predators (metanalysis)

Diet information was collected from published literature on the gut contents of piscivorous coral reef teleost fishes in the Indo-Pacific realm (S1 Fig). Information extracted from the literature was: Range of predator body sizes sampled from each study, predator species, prey species, and number of occurrences that the prey species was found in predator guts. Prey species were then classified into functional groups (as above). We removed pelagic predators as they likely operate at a broader spatial scale than more benthic associated predators.

Individual body sizes were not available, as only size ranges were reported in the literature. Therefore, for every predation event recorded from the literature, we drew individual body sizes from a uniform distribution delimited by the range of sizes provided by the respective study. This process was done for each predation event recorded (*n* = 1,224) and was simulated 1,000 times with repetition. In some instances, the exact size of the predator was recorded, and was therefore used as the only potential body size for the given predation events, throughout the simulations. These observed predation events were compared to our simulated predation events based on the overlap coefficient from the R-package “bayestestR” [72]. These predation events were then assigned into the same body size bins as the ones used in our community survey (see above). We then summarised the relative contribution of each prey functional group to each predator body size bin (as above).

## Supporting information

S1 Fig Map showing the sites from which studies of our metanalysis conducted gut content analyses. Original map downloaded from Natural Earth (www.naturalearth.com/downloads).
(DOCX)

S2 Fig (a) Current and (b) suggested models of body size vs. mortality relationships. While the overall mortality remains the same between the current and suggested model (area below curves), the shape of the suggested distribution (i.e., flattening the curve), results in different survivorship curves (c, d). These differences in survivorship curves may allow for a higher number of reproducing individuals and provide a potential explanation for limited individual gamete output, resulting in high overall contribution to the larval pool near coral reefs (Brandl and colleagues [20]). Fish silhouettes redrawn from (Mihalitsis and colleagues [36]).
(DOCX)

S1 Table Functional groups used in our study, along with a description of their functional traits directly related to predator–prey relationships. (DOCX)
