# Supplementary material for: Small predators dominate fish predation in coral reef communities
Source: PLoS Biol. 2022 Nov 29;20(11):e3001898. doi: 10.1371/journal.pbio.3001898 (PMC9707750; doi:10.1371/journal.pbio.3001898)
Supplement: S1 Fig — Original map downloaded from Natural Earth (www.naturalearth.com/downloads). (DOCX) [file pbio.3001898.s001.docx]

**Supplemental Material**


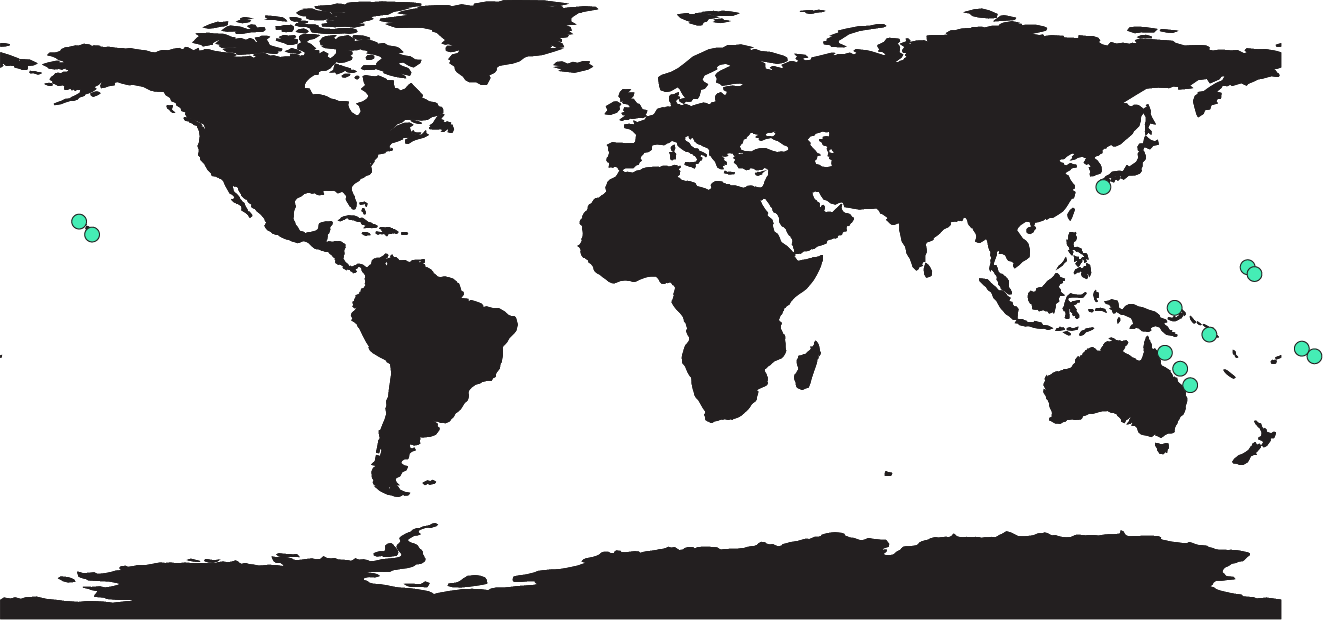


**Supplemental Figure 1**. Map showing the sites from which studies of our metanalysis conducted gut content analyses. Original map downloaded from Natural Earth ([www.naturalearth.com/downloads](http://www.naturalearth.com/downloads)).

Public domain: <https://www.naturalearthdata.com/about/terms-of-use/>
