# Supplementary material for: Small predators dominate fish predation in coral reef communities
Source: PLoS Biol. 2022 Nov 29;20(11):e3001898. doi: 10.1371/journal.pbio.3001898 (PMC9707750; doi:10.1371/journal.pbio.3001898)
Supplement: S2 Fig — (a) Current and (b) suggested models of body size vs. mortality relationships. While the overall mortality remains the same between the current and suggested model (area below curves), the shape of the suggested distribution (i.e., flattening the curve), results in different survivorship curves (c, d). These differences in survivorship curves may allow for a higher number of reproducing individuals and provide a potential explanation for limited individual gamete output, resulting in high overall contribution to the larval pool near coral reefs (Brandl and colleagues [20]). Fish silhouettes redrawn from (Mihalitsis and colleagues [36]). (DOCX) [file pbio.3001898.s002.docx]

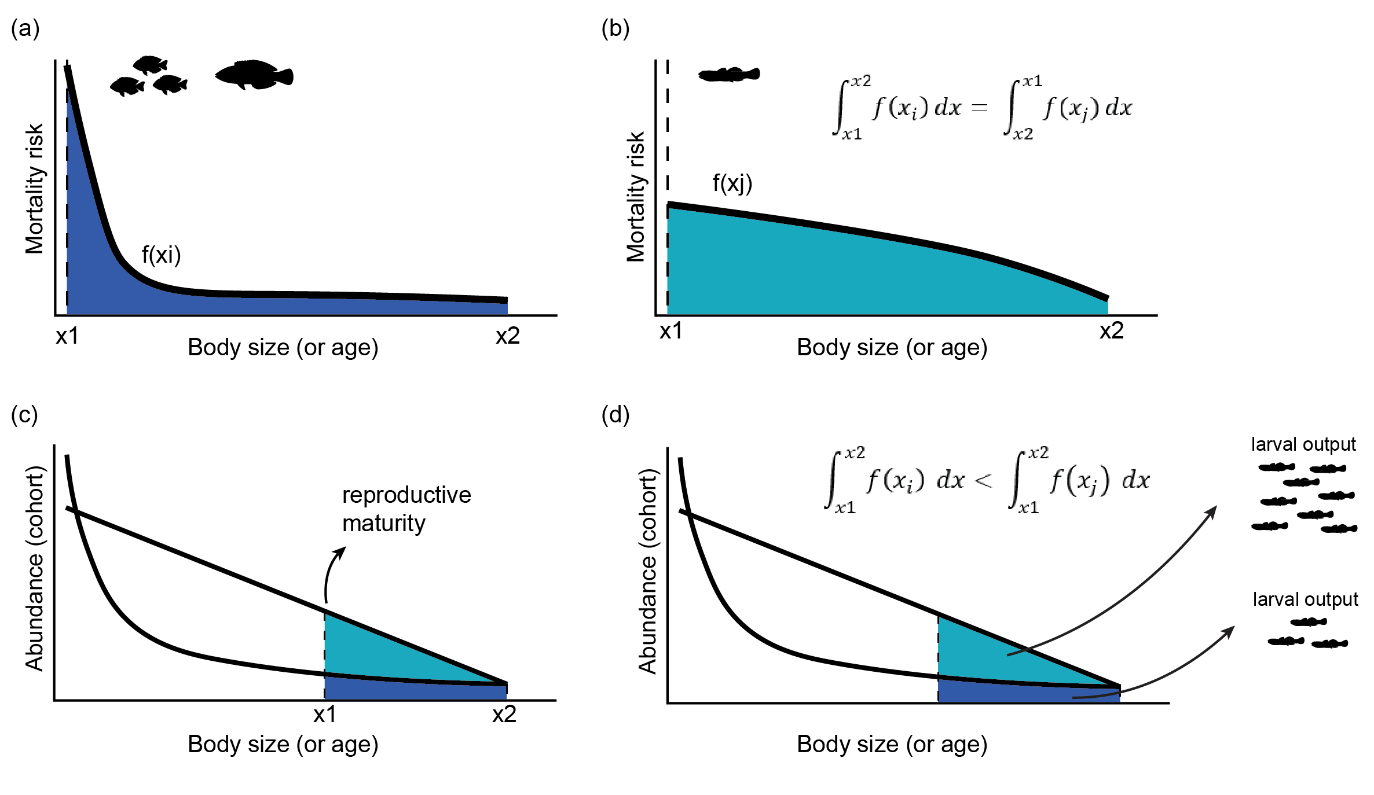


**S2 Fig**. (a) Current and (b) suggested models of body size vs. mortality relationships. While the overall mortality remains the same between the current and suggested model (area below curves), the shape of the suggested distribution (i.e., flattening the curve), results in different survivorship curves (c-d). These differences in survivorship curves may allow for a higher number of reproducing individuals, and provide a potential explanation for limited individual gamete output, resulting in high overall contribution to the larval pool near coral reefs (Brandl *et al.* 2019). Fish silhouettes redrawn from (Mihalitsis *et al.* 2021).

**References**

1.Brandl, S.J., Tornabene, L., Goatley, C.H.R., Casey, J.M., Morais, R.A., Côté, I.M. *et al.* (2019). Demographic dynamics of the smallest marine vertebrates fuel coral reef ecosystem functioning. *Science*.

2.Mihalitsis, M., Hemingson, C.R., Goatley, C.H. & Bellwood, D.R. (2021). The role of fishes as food: A functional perspective on predator‐prey interactions. *Functional Ecology*, 35, 1109-1119.
