# Supplementary material for: Small predators dominate fish predation in coral reef communities
Source: PLoS Biol. 2022 Nov 29;20(11):e3001898. doi: 10.1371/journal.pbio.3001898 (PMC9707750; doi:10.1371/journal.pbio.3001898)
Supplement: S1 Table — (DOCX) [file pbio.3001898.s003.docx]

|  | **Functional group** | **Description** |
| --- | --- | --- |
| **Predator** | Grabber | macrodont dentition (Mihalitsis & Bellwood 2019) with relatively large jaw musculature. Can strike from larger distances (>2 body lengths). Captures prey by grabbing it tail- or body-first, usually followed by headshaking behaviour. |
|  | Engulfer | villiform or edentulate dentition (Mihalitsis & Bellwood 2019) with relatively small jaw musculature. Strikes from high angles (above or below prey) and relatively small distances. Captures prey primarily by engulfing it whole. |
| **Prey** | Social | primarily deep bodied schooling planktivores swimming higher up in the water column. |
|  | Epibenthic | more elongate, solitary, and swimming above, but closer to the benthos |
|  | Cryptobenthic | primarily elongate, solitary, and 'sitting' on the benthos |

**S1Table**. Functional groups used in our study, along with a description of their functional traits directly related to predator-prey relationships.

**References**

1.Mihalitsis, M. & Bellwood, D.R. (2019). Functional implications of dentition-based morphotypes in piscivorous fishes. *Royal Society open science*, 6(9), 190040.
